# Ethanol Extracts of Rice Bran and Whole Grain Adlay Seeds Mitigate Colonic Inflammation and Damage in Mice with Colitis

**DOI:** 10.3390/nu14183877

**Published:** 2022-09-19

**Authors:** Hui-Chen Lo, Yu-Hsin Chen, Wen-Tzu Wu

**Affiliations:** 1 Department of Nutritional Science, Fu Jen Catholic University, New Taipei City 242062, Taiwan; 2 Taichung District Agricultural Research and Extension Station, Tatsuen Hsiang, Changhua County 515008, Taiwan; 3 Department of Food Nutrition and Health Biotechnology, Asia University, Taichung 41354, Taiwan

**Keywords:** ulcerative colitis, rice bran, whole-grain adlay seeds, cytokines, tight junction, oxidative stress

## Abstract

Ulcerative colitis (UC) is a chronic inflammatory bowel disease with frequent relapsing inflammation in the colon. Whole grains have been promoted as healthy and sustainable foods; however, the use of whole gains in UC is inconclusive. The aim of this study was to investigate the effects of ethanol extracts of rice bran (RBE) and whole-grain adlay seeds (ADE) on inflammation, oxidative stress, and colonic damage in UC. Male C57BL/6JNarl mice were intra-rectal injected twice with 2,4-dinitrobenzene sulfonic acid to induce (day 0) and reactivate (day 21) UC. Control mice were fed AIN-93M diet (R group) and injected with a vehicle. UC mice were fed AIN-93M diet (UC group) supplemented with RBE (RBE group) or ADE (ADE group) for 21 days. The results showed that the UC group had an increased disease activity index, plasma interleukin (IL)-6 and glutathione levels, microscopic injury scores, and inflammatory cytokine and chemokine levels in the colon and decreased colonic claudin-4 compared to the R group. RBE and ADE supplementation significantly reduced UC-elevated plasma IL-6 and colonic glutathione and pro-inflammatory cytokines and a chemokine. In addition, RBE and ADE supplementation significantly decreased T-helper-cell-associated cytokines in the plasma and colon. Moreover, RBE supplementation increased colonic IL-10 and tight junction protein claudin-4 levels, and ADE supplementation alleviated diarrhea in UC mice. In conclusion, these results suggest that RBE and ADE may mitigate colonic inflammation, oxidative stress, and damage in UC relapse.

## 1. Introduction

Ulcerative colitis (UC), a subtype of inflammatory bowel disease (IBD), is a chronic, relapsing inflammatory disease that occurs primarily in the rectum and colon in a continuous pattern. The clinical symptoms of UC, including episodes of diarrhea, fever, fatigue, abdominal pain, cramping, bloody stool, reduced appetite, and weight loss, result in a significant decrease in quality of life and social functioning of patients. In addition, the presence of discontinuous and recurring inflammatory lesions in the rectal and colonic mucosa may increase the risk of colorectal cancer in UC patients [1]. The incidence and prevalence of UC are high in Europe and North America and are increasing in Asian countries [2]. In Taiwan, the prevalence rates of UC have increased more than six-fold in the past 20 years [2], and this increase has occurred predominantly in males [3].

UC is an autoimmune disease; however, its etiology remains unknown. Genetic influences, dysregulation of mucosal immunological barrier function, and overproduction of pro-inflammatory cytokines and reactive oxygen species (ROS) have been found to contribute to colonic tissue damage and ulceration [4]. Western diets that contain high amounts of fats, saccharides, and refined sugar with low dietary fiber content are considered one of the environmental factors that induce the onset and relapse of UC [5]. Diets that are rich in fruits, vegetables, and whole grains may be used to decrease the recurrence of IBD and the risk of colon cancer [6]. However, a diet composed of highly fermentable oligo-, di-, monosaccharides and polyols (FODMAP) may not be appropriate for UC [7]. Indeed, a cross-sectional study indicated that a modified diet containing approximately two-thirds of the daily recommendation in fiber, whole grains, fruits, and vegetables may reduce IBD symptoms [8].

Recent studies have shown that nutraceuticals with antioxidant and anti-inflammatory activities may have beneficial effects on IBD [9]. Whole grains contain the bran, germ, and endosperm, which provide various health-promoting nutrients, such as vitamins, minerals, fibers, and phytochemicals [8,10,11,12]. Rice bran, a nutrient-rich byproduct of the rice-milling process, has been considered a sustainable source of functional ingredients [13]. Its bioactive components—mainly phenolic and flavonoid compounds and vitamin E—have been found to have antioxidant, anti-inflammatory, anti-diabetic, and anticancer activities [14,15]. In rats, pretreatment of rice bran ethanol extracts significantly alleviated lipopolysaccharide-induced oxidative stress [13]. In mice with dextran sodium sulfate-induced UC, dietary supplementation of fermented rice bran alleviated mucosal inflammation; crypt loss; inflammatory cell infiltration; myeloperoxidase activity; thiobarbituric acid reactive substance (TBARS) levels; and T-helper-cell-associated cytokines, such as interleukin (IL)-1β, IL-6, and IL-17 [9,16]. The attenuated intestinal inflammation and enhanced tight junction barrier integrity suggest that rice bran may effectively protect against inflammation-induced intestinal fibrosis [16].

The adlay seed (*Coix lacryma-jobi* L.)—mainly the polished seed without bran—is an annual crop that is traditionally used as a medicinal food. Evidence has shown that the fraction with high phenolic and flavonoid contents from the ethanol extracts of adlay bran has an anti-inflammatory effect [12]. Polysaccharides and phenolic compounds extracted from adlay, especially the adlay bran, by ethanol, ethyl acetate, or acidified methanol have anti-inflammatory, immunological, and anti-hyperuricemia activities [11,17,18,19] and may improve epithelial barrier dysfunction in Caco-2 cells [11]. The anti-inflammatory and anti-apoptotic effects of ethanol extracts of the adlay hull have been demonstrated in rat brain PC-12 cells [20]. These results reveal that whole-grain adlay seeds may have beneficial effects for UC patients.

In clinical practice, no primary nutrition therapy is available for UC patients in the acute phase [5]. In this study, extracts of rice bran and whole-grain adlay seeds were created by using ethanol instead of water for extraction to exclude the water-soluble dietary fiber and to obtain higher yields of phenolic compounds. Here, mice with dinitrobenzene sulfonic acid (DNBS)-induced UC were used to evaluate the efficacy of these extracts on alleviating the disease activity index (DAI) and UC-induced alterations in plasma and colonic cytokine levels, macroscopic and microscopic injury, collagen levels, antioxidant status, and tight junction proteins in the colon. We hypothesized that ethanol extracts of rice bran (RBE) and whole-grain adlay seeds (ADE) may have antioxidant and anti-inflammatory activities that can alleviate the severity of UC.

## 2. Materials and Methods

### 2.1. Animals

The protocols and animal procedures were reviewed and approved by the Institutional Animal Care and Use Committee of Fu-Jen Catholic University (#A10803). Male C57BL/6JNarl mice at 6 weeks old (20 to 22 g) were purchased from the National Laboratory Animal Center (Taipei, Taiwan) and were housed in a room maintained at 22 °C on a 12:12 h light–dark cycle with free access to water and a normal chow diet (5001 Laboratory Rodent diet, Labdiet^®^, Richmond, IN, USA). After 2 weeks of acclimation, mice were divided into normal, healthy (R group, n = 10), and UC (3 groups, n = 12/group) groups and adapted with semi-purified AIN-93M diets by gradually introducing the diet over a 3-day period.

### 2.2. Ethanol Extract Preparations and Experimental Diets

Rice bran (stabilized, Herbfood Co., Taichung, Taiwan) and whole-grain adlay seeds (Taichung No. 5’ job’s tears, *Coix lacryma-jobi* L.) were extracted with 70% ethanol, as described in the study of Chen et al. [21]. In brief, the powders of rice bran and whole-grain adlay seeds were defatted twice with hexane. Subsequently, the defatted materials were extracted with 10-fold volume of 70% ethanol overnight, at 4 °C, with shaking. After centrifugation at 10,000×*g* at 4 °C for 15 min, the supernatant was collected, filtered, ethanol-evaporated, and lyophilized. The dry extract was stored at −20 °C for further use.

The doses of ethanol extracts were calculated from the recommended daily doses of whole-grain brown rice (100 g per day, containing 16% of weight as rice bran) and whole-grain adlay seeds (50 g per day) for human adults. In the rice-milling process, approximately 16 g of rice bran was obtained from 100 g of whole-grain brown rice, and approximately 1.2 g of ethanol extract was obtained from the rice bran. In addition, approximately 0.64 g of ethanol extract was obtained from 50 g of whole-grain adlay seeds.

To prepare the experimental diets with ethanol extracts, the mouse metabolic rate was estimated based on the human metabolic rate, assuming a 12.3-fold difference. The average daily food intake was approximately 4 g for a mouse weighing 25 g. Therefore, 1.5 and 0.8 g of ethanol extracts from rice bran and whole-grain adlay seeds, respectively, were added into 1 kg of semi-purified AIN-93M diet, as shown in Table 1. All diet ingredients were purchased from Dyets Inc. (Bethlehem, PA, USA). The three different diets provided identical amounts of calories, protein, fat, fiber, minerals, and vitamins, with the exception of vitamin E.

### 2.3. Induction of UC

UC induction methods were based on the description by Martin et al. [22]. Following an overnight fast, mice in the UC, RBE, and ADE groups were anesthetized with 3% isoflurane (day 0) and intra-rectally injected with 200 mg/kg of 2,4-dinitrobenzene sulfonic acid (DNBS in 50% ethanol, Sigma, St. Louis, MO, USA) via PE-50 tubes. Mice in the R group were injected with 100 μL of 50% ethanol to control for the stress from the intra-rectal injection. After the injection, 2% agar gel with 6% sucrose was provided for 3 days to prevent the occurrence of hypoglycemia and dehydration. On day 21, UC was reactivated with 100 mg/kg of DNBS. All mice were sacrificed on day 24, and blood samples were collected by cardiac puncture for the collection of red blood cells and plasma. The colon was dissected, weighed, and collected for further analysis.

### 2.4. Experimental Design

From day 3 to day 24, the UC mice were fed semi-purified AIN-93M diets with or without different ethanol extracts. UC mice were divided into three groups and fed either the AIN-93M diet (UC group) or the AIN-93M diet supplemented with 70% alcohol extract of rice bran (RBE group) or whole-grain adlay seeds (ADE group).

### 2.5. Measurements

#### 2.5.1. Body Weight, Food Intake, and Disease Activity Index (DAI)

During the experimental period, body weight was recorded twice per week, and food intake, stool consistency, and signs of hematochezia were observed daily. The DAI was calculated by using scores correlating with body weight loss, diarrhea, and hematochezia [23]. Stool consistency was graded by using the following scale: 0 for normal stool, 2 for loosely stool, and 4 for diarrhea. The hematochezia score was graded as 0 for no blood being seen, 1 for possible bleeding undetectable by the naked eye, 2 for visible bleeding, and 4 for hematochezia around the anus. The body weight loss score was graded as 0 for no weight loss, 1 for 1–5% of total body weight loss, 2 for 5–10%, 3 for 10–20%, and 4 for over 20% of total body weight loss. The DAI was then calculated by using the following equation. DAI = (body weight lost score + diarrhea score + hematochezia sore)/3.

#### 2.5.2. Inflammatory Mediators in the Plasma and Colon

To evaluate the effects of the ethanol extracts on systemic and local inflammation, plasma and colonic cytokines, including the T-helper-cell-associated tumor necrosis factor (TNF)-α, IL-6, interferon (IFN)-γ, IL-10, and IL-12p70, were measured. DNBS-induced colitis is particularly useful in studying T-cell-dependent immune mechanisms [24]. Levels of inflammatory mediators and the chemokine monocyte chemoattractant protein-1 (MCP-1) were determined by using commercially available cytometric bead assays (BD Biosciences, Minneapolis) and analyzed on a flow cytometer (BD Accuri^®^ C6, Becton, Dickinson and Company, Franklin Lakes, NJ, USA).

#### 2.5.3. Macroscopic Injury Score of the Colon

After the mice were euthanized, the total colon length was recorded, and any injury was photographed with a digital camera (Coolplex 4500, Nikon, Tokyo, Japan). The macroscopic injury score was based on the study of Impellizzeri et al. [25]. The scoring system was graded as the following: 0 for no visible damage; 1 for small hyperemia without ulcers; 2 for linear ulcers and no major inflammation; 3 for linear ulcers with one site of inflammation; 4 for two or more sites of inflammation with ulceration covering >1 cm along the length of the colon; and 5 for two or more major sites of inflammation and ulceration or one major site of inflammation and ulceration extending >2 cm along the length of the colon and an additional point for each centimeter of ulceration beyond the initial 2 cm [25].

#### 2.5.4. Collagenous Connective Tissue of the Colon: Masson’s Trichrome Stain

After colon collection, 1 cm of colon from the region approximately 3 cm proximal to the distal segment of the colon was collected and fixed in 10% buffered formalin for routine paraffin embedding [24]. The 4 μm paraffin-embedded sections were stained with hematoxylin and eosin (H&E). Histological changes of colonic sections, including the depth of ulceration, area of ulceration, edema, and immune cell infiltration, were scored by using a graded scale of 0 to 4, as described by Iwazawa et al. [26]. In addition, the severity of inflammation, hyperplasia, and the loss of goblet cells in the mucosa were graded according to the modified method described by Impellizzeri et al. [25] and Shackelford et al. [27]. The interpretation of these scores was performed by an animal pathologist (National Taiwan University College of Medicine Laboratory Animal Center, Taipei, Taiwan) with an official pathology report.

#### 2.5.5. Histopathological (Microscopic) Assessment of the Colon

To detect collagen fiber deposition, 4 µm colonic sections were stained with a Masson’s trichrome staining kit (TRM-1-IFU, Logan, UT, USA), according to the manufacturer’s instruction. In brief, the deparaffinized slides were incubated in preheated Bouin’s fluid for 60 min, then cooled, rinsed, and stained with Weigert’s iron hematoxylin. Next, the slides were incubated with Biebrich scarlet-acid fuchsin solution and differentiated in phosphomolybdic-phosphotungstic acid solution. Subsequently, 1% acetic acid solution was applied to the slides, which were then dehydrated with 95% alcohol and absolute alcohol, cleared in xylene, and mounted in synthetic resin. The immunoreaction was observed by using a light microscope (Eclipes TE2000-U; Nikon, Tokyo, Japan). The area of blue color, representing collagen, was semi-quantified in five fields per slide at 200× magnification, using Image-Pro Plus software version 6.0 (Media Cybernetics; Rockville, MD, USA). The ratio of collagen in each field was calculated as the blue area (collagen) divided by the total red (muscle fiber), blue (collagen), and dark-red-to-black (nuclei) areas combined.

#### 2.5.6. Tight Junction Proteins in the Colon

To further investigate gut integrity at the molecular level, two transmembrane tight junction proteins, claudin-4 and occludin, were measured by using immunohistochemistry (IHC) analysis. After deparaffinization and dehydration, 4 μm colonic sections were treated with 10 mM sodium citrate at 100 °C for 15 min for antigen retrieval, 3% H_2_O_2_ for 10 min for quenching endogenous peroxidase, and 5% skim milk for 60 min for nonspecific blocking, followed by hybridization with the primary antibodies against claudin-4 (1:500, Thermo Scientific, Waltham, MA, USA) and occludin (1:50, Santa Cruz, Dallas, TX, USA). Horseradish peroxidase and rabbit anti-mouse biotinylated IgG (1:200), diaminobenzidine substrate stain, and hematoxylin were used as per the manufacturer’s protocol (Thermo Scientific, Waltham, MA, USA). Immunoreactions were observed by using a light microscope (Nikon, Tokyo, Japan) with a cooling-charge-coupled-device camera. Semi-quantitative analysis of the images was performed in five fields per slide (Image-Pro Plus software version 6.0). The ratio of claudin-4 and occludin to total colonic area in each field was calculated.

#### 2.5.7. Indicators of Oxidative Stress in the Colon

The levels of lipid peroxidation products, TBARS, were determined in the colon, using the methods presented by Ohkawa et al. [28]. Non-enzymatic antioxidants, i.e., reduced glutathione (GSH) and oxidized glutathione (GSSG), were measured fluorometrically, according to the methods of Hissin and Hilf [29]. The ratio of GSH to GSSG was calculated to evaluate oxidative status.

### 2.6. Statistical Analysis

Values are reported as the mean ± the standard error of the mean (SEM) for continuous measures and as the median and interquartile range (IQR) for nonparametric measures. For continuous measures, all groups were compared by one-way analysis of variance (ANOVA), using the SAS general linear model program (SAS Institute, Cary, NC, USA). Group means were considered significantly different at *p* < 0.05. The Duncan's multiple range test was used as a post hoc analysis to compare the differences among the groups when the ANOVA indicated an overall significant group effect, *p* < 0.05. The Kruskal–Wallis test was used for the analyses of macroscopic and microscopic injury scores, stool consistency, and signs of hematochezia, followed by the Mann–Whitney U test to determine significant differences (*p* < 0.05) between groups

## 3. Results

### 3.1. Food Intake, Body Weight, DAI, and Colon Length

**Figure 1:**
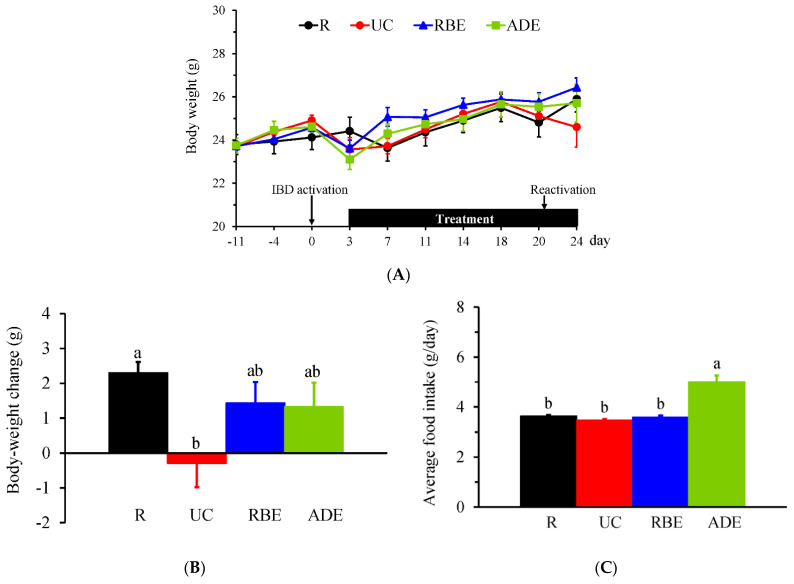
Body weight and food intake of control mice (R group) fed AIN-93M diet and UC mice fed either the AIN-93M diet (UC group) or the AIN-93M diet supplemented with alcohol extract of rice bran (RBE group) or whole-grain adlay seeds (ADE group). (**A**) Body weight before and on the day of UC induction (day 0) until the end of the experiment (day 24). (**B**) Body-weight change and (**C**) average daily food intake from day 0 to day 24. Values are mean ± SEM, n = 10−11 per group. Different superscript letters indicate significant differences (one-way ANOVA and Duncan’s multiple range test, *p* < 0.05).

The final sample size for each group was 10 mice in the R, UC, and RBE groups and 11 mice in the ADE group. After the intra-rectal injection of DNBS, five mice died within 2 days. The autopsy confirmed that the cause of death was large bowel perforation. During the experimental period, the body weight (Figure 1A) was not significantly different among groups, but the body-weight change (Figure 1B) from day 0 to day 24 was significantly lower in the UC group compared to the R group. The average daily food intake during the experimental period was not significantly different between the R and UC groups (Figure 1C). However, the ADE group had significantly greater food intake than the R, UC, and RBE groups.

Diarrhea (stool consistency), bloody stool (hematochezia) and DAI scores and the length of the colon on day 24 are shown in Table 2. The UC group had significantly higher diarrhea, bloody stool, and DAI scores than the R group. The ADE group had a significantly decreased diarrhea score compared to the UC group. A shortened colon length is one of the signs of IBD; however, the colon length was not significantly different among groups.

### 3.2. Inflammatory Mediators in the Plasma

There were no significant differences in the levels of TNF-α, IFN-γ, IL-10, IL-12p70, or MCP-1 between the R and UC groups (Table 3). The UC group had significantly increased IL-6 levels compared to the R group, and the RBE and ADE groups had significantly decreased IL-6 levels compared to the UC group. In addition, the RBE and ADE groups had significantly decreased the levels of TNF-α, IFN-γ, IL-10, IL-12p70, and MCP-1 compared to the R and UC groups (Table 3).

### 3.3. Macroscopic and Microscopic Injury Scores of the Colon

The macroscopic injury scores, including hyperemia, ulcers, and inflammation, were used to evaluate colon damage. There were no significant differences in the colonic macroscopic injury scores among groups. To evaluate microscopic injury, colonic sections were stained with H&E. The scores of edema, immune-cell infiltration, loss of goblet cells, inflammation, and total injury in the colon were significantly increased in the UC, RBE, and ADE groups compared to the R group (Table 4); however, there were no significant differences among the UC, RBE, and ADE groups. In addition, there were no significant differences in the depth of ulceration, area of ulceration, and hyperplasia scores in the colon among the groups.

### 3.4. Collagen, Claudin-4, and Occludin Contents in the Colon

**Figure 2:**
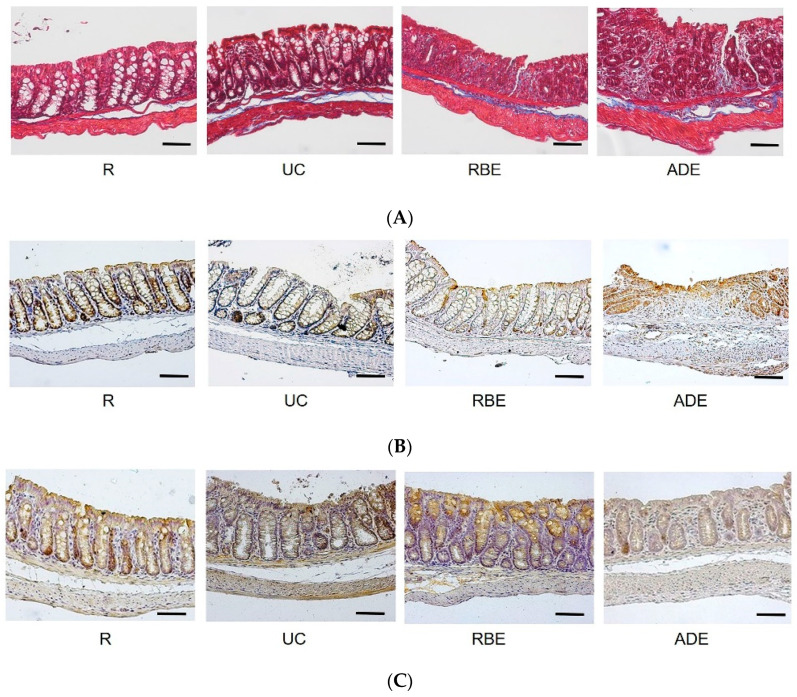

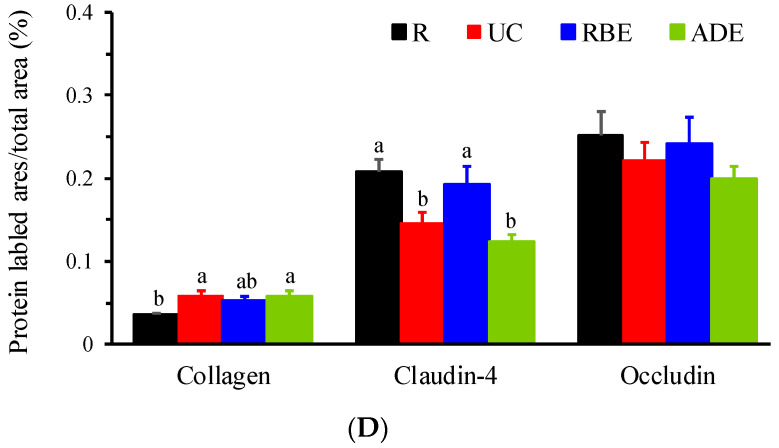
Protein expression of collagen, claudin-4 and occludin in the colon of control mice (R group) fed AIN-93M diet and UC mice fed either the AIN-93M diet (UC group) or the AIN-93M diet supplemented with alcohol extract of rice bran (RBE group) or whole-grain adlay seeds (ADE group). Photos (100×, bars = 50 μm) of Masson’s trichrome staining for (**A**) collagen, and immunohistochemical staining of (**B**) claudin-4 and (**C**) occludin in the colon and (**D**) the ratio of indicated protein area to total colonic area. Values are mean ± SEM, n = 10−11 per group. Different superscript letters represent significant differences (one-way ANOVA and Duncan's multiple range test, *p* < 0.05).

To evaluate the degree of colonic damage, Masson's trichrome staining was used to detect collagen deposition, i.e., an index of fibrosis. As shown in Figure 2A, the blue color represents collagen, the red represents muscle fibers, and the dark-red-to-black areas represent nuclei. The protein expression of tight junction proteins, i.e., claudin-4 (Figure 2B) and occludin (Figure 2C), was measured by IHC staining with brown color in the colonic sections. When calculating the area of collagen in total colonic area of the microscopic field, the UC and ADE groups had a significantly greater collagen-positive area than the R group (Figure 2D). Moreover, the UC and ADE groups had a significantly decreased proportion of claudin-4 compared to the R and RBE groups (Figure 2D). However, the occludin-positive area was not significantly different among groups.

### 3.5. GSH, GSSG and TBARS Levels in the Colon

Levels of antioxidant indicators, GSH, GSSG, the ratio of GSH to GSSG, and lipid peroxidation indicators (TBARS) in the colon are shown in Table 5. The UC group had significantly increased GSH levels and a higher GSH:GSSG compared to the R, RBE, and ADE groups. There were no significant differences in GSSG and TBARS levels in the colon among groups.

### 3.6. Inflammatory Mediators in the Colon

Cytokine and chemokine levels in the colon are shown in Table 6. The UC group had approximately 0.8- to 1.5-fold higher TNF-α, IL-6, IFN-γ, IL-12p70, and MCP-1 levels compared to the R group. In contrast, IL-10 in the colon of the UC group was 50% lower than that in the R group. The UC-induced increases in colonic levels of TNF-α and IL-12p70 were significantly decreased in the RBE and ADE groups. Furthermore, the UC-induced increases in IL-6 and MCP-1 and the UC-induced decrease in IL-10 were significantly ameliorated in the RBE group.

## 4. Discussion

The prevalence of UC is gradually increasing in Taiwan. Its symptoms and relapses threaten the quality of life and increase the risk of colorectal cancer in UC patients [30]. In clinical practice, UC treatment includes corticosteroids, antibiotics, and immunosuppressants; however, none of these drugs are curative, and their long-term use may result in adverse side effects and complications [31]. Foods with antioxidant, anti-inflammatory, and immunomodulatory activities, such as whole grains, are considered to have beneficial effects for UC patients [8,32]. In the present study using DNBS-induced UC mice, oral administration of ethanol extracts from rice bran and whole-grain adlay seeds significantly alleviated the DNBS-induced alterations in T-helper-cell-associated cytokines. In addition, the ethanol extracts of rice bran significantly improved DNBS-induced decreases in the tight junction protein claudin-4’s levels in the colon.

Increasing evidence suggests that inappropriate food choice, for instance, a Western diet, may induce the overproduction of pro-inflammatory cytokines and dysregulate immune function, resulting in recurrence, delayed recovery, or worsening disease severity in UC patients [9]. In addition, a low FODMAP diet is recommended for UC patients during the active period to control gastrointestinal symptoms [32,33]. Whole grains, for example, rice bran and adlay, are traditional Chinese foods that have been demonstrated to have antioxidant and anti-inflammatory activities [9,12,16,18,34]. However, the high dietary fiber content in whole grains may limit their use in UC patients. After defatting and excluding polysaccharides, starch, and fiber, ethanol extracts of rice bran and whole-grain adlay seeds contain copious amounts of phenolic and flavonoid compounds and vitamin E. These materials may have potential biological functions in UC.

DNBS is a hapten that can trigger the host innate and adaptive immune responses and has been used in mice to mimic the relapsing pattern observed in UC patients [22,24]. In our previous study, DNBS-induced UC mice were administered various water extracts derived from grains and plants, including *Echinacea purpurea*, *Salvia miltiorrhiza*, and adlay polysaccharides; however, clinical symptoms and colon damage in UC mice were not improved [35]. We speculated that the high content of dietary fiber from these water extracts might not be appropriate for UC mice during the relapsed state. Thus, the present study focused on rice bran and whole-grain adlay seeds that were extracted with 70% ethanol to reduce FODMAP and further evaluate their effects on colonic injury and inflammation.

In the present study, body weight was significantly reduced in UC mice during the experimental period, and DAI and microscopic injury scores, including edema, immune cell infiltration, goblet cell loss, inflammation, and total injury in the colon, were increased. In addition, UC mice had significantly increased plasma IL-6 levels, elevated colonic pro-inflammatory and T-helper-cell-associated cytokines and chemokine MCP-1 levels, and a decreased anti-inflammatory cytokine IL-10 level. Tight junctions act as a semipermeable barrier and function to divide the apical and basolateral domains of the plasma membrane [36]. Collagen, an index of fibrosis, has been shown to deposit in the colon from the mucosal to the muscular layers in UC patients [37]. In the present study, UC mice had a significant decrease in the levels of the tight junction protein claudin-4 and a significant increase in collagen in the colon. The changes in clinical symptoms, microscopic colonic injury, cytokine profiles in the plasma and colon, tight junction protein, and collagen reveal that the DNBS-induced UC mice had similar clinical and histological features to UC patients.

GSH, a major antioxidant in the body, contains a free thiol group that can be oxidized to GSSG. In the present study, UC mice had unaltered colonic TBARS and increased colonic GSH levels, suggesting an adaptive response to alleviate oxidative stress, such as lipid peroxidation, in the colon during UC reactivation. The low DAI scores and unchanged colonic length, colonic TBARS, and plasma proinflammatory cytokines suggest that the DNBS-induced UC mice may have mild-to-moderate disease severity and colon damage, which were not as severe as the findings in the study of Martin et al. [22]

Whole grains are a good source of dietary fiber, minerals, vitamins, and phytochemicals. Rice bran, a by-product of rice milling, has drawn attention due to its surplus value. In human umbilical vein endothelial cells, rice-bran-derived phenolic extracts showed antioxidant and anti-inflammatory effects that protected against endothelial dysfunction [10]. In a randomized double-blind controlled trial, γ-oryzanol, a triterpenic alcohol mixture with ferulic acid esters and phytosterols extracted from rice bran, improved antioxidant status in hyperlipidemic subjects [14,38]. The antioxidant and anti-inflammatory effects of rice bran components, including oligosaccharides, γ-oryzanol, ferulic acid, geranylgeraniol, farnesol, isoprenoids, and lectins, have been reviewed [15,39]. In the present study, an ethanol extract of rice bran significantly decreased plasma and colonic levels of IL-6; T-helper-cell-associated cytokines, such as TNF-α and IL-12p70; and the chemokine MCP-1, a marker secreted by monocytes, memory T cells, and dendritic cells in UC mice [9,16,40]. In addition, the ethanol extract of rice bran significantly increased claudin-4 protein expression and alleviated UC-elevated GSH content in the colon. The decreased GSH and unaltered TBARS reveal that an ethanol extract of rice bran may reduce the need for high levels of antioxidants, e.g., GSH, to maintain the colonic redox status in UC. The results of plasma and colonic cytokines and the chemokine MCP-1 and colonic claudin-4 and GSH content suggest that the ethanol extract of rice bran may alleviate UC-induced impairment in colonic integrity, inflammation, and oxidative stress.

Adlay seeds, also called Job’s tear seeds or Coix seeds, have been used as nourishment foods and in traditional Chinese medicine and possess antioxidant and anti-inflammatory activities [18,34]. In Caco-2 cells, polysaccharides of adlay bran significantly decreased the secretion of pro-inflammatory cytokines and IL-10 and alleviated TNF-α-induced epithelial barrier dysfunction [11]. In a rat model of rheumatoid arthritis, an ethanol extract of adlay seed significantly decreased serum pro-inflammatory cytokines and increased the activities of antioxidant enzymes [34]. In the present study, UC mice administered the ethanol extract from whole-grain adlay seeds had an approximately 25% increase in food intake without subsequent weight gain, suggesting a potential role for adlay seed extract in weight control. In addition, diarrhea; T-helper-cell-associated cytokines in the plasma; and DNBS-induced increases in TNF-α, IL-12p70, and GSH in the colon were decreased by dietary supplementation with an ethanol extract of whole-grain adlay seeds in UC mice. These results suggest that an ethanol extract of whole-grain adlay seeds may be used to alleviate clinical symptoms and colonic inflammation, at least partially, in UC patients.

There are several limitations in the present study. First, components of the ethanol extracts were not determined, thus limiting the ability to distinguish the main bioactive compound(s) in rice bran and whole-grain adlay seeds. Second, the severity of the UC induced in the study was mild to moderate, so that the UC mice would present an adaptive redox response, such as unaltered TBARS and increased GSH levels in the colon. This adaptive response limited the degree of improvement observed in oxidative-stress markers in UC mice administered the ethanol extracts. Third, the mucosal and muscular layers of the colon were not separated when determining cytokine and chemokine levels. The mucosal layer is the major site with immunological processes in the colon. Therefore, the cytokines, chemokine MCP-1, GSH, and TBARS results in the colon may be underestimated. Even so, cytokine and chemokine levels were simultaneously determined in the plasma and colon, allowing us to reveal that the systemic and local inflammatory and immune responses were different in UC.

## 5. Conclusions

Our findings suggest that oral administration of ethanol extracts from rice bran and whole-grain adlay seeds may partially alleviate the clinical symptoms, morphological changes of the colon, and colonic damage in DNBS-induced UC mice. In addition, these ethanol extracts may eliminate DNBS-induced alterations in T-helper-cell-associated cytokines and GSH in the colon. Furthermore, the ethanol extract of rice bran has beneficial effects in regard to preserving colon integrity via increasing the tight junction protein claudin-4 in UC mice. The bioactive components and mechanisms of ethanol extracts from rice bran and whole-grain adlay seeds in modulating the homeostasis of inflammatory mediators and colonic oxidant–antioxidant profiles in UC, as well as the safety and tolerability of these extracts, are worth further investigation.

## Figures and Tables

**Table 1:** Composition of AIN-93M-modified diets.

Ingredient (g)	R/UC Groups	RBE Group	ADE Group
Corn starch	465.7	464.2	464.9
Rice bran extract	0	1.5	0
Whole-grain adlay seeds’ extract	0	0	0.8
Dextrin	155	155	155
Casein, vitamin-free	140	140	140
Sucrose, granular	100	100	100
Cellulose	50	50	50
Soybean oil	40	40	40
AIN-93M Mineral Mix	35	35	35
AIN-93M Vitamin Mix	10	10	10
Choline bitartrate	2.5	2.5	2.5
L-cystine	1.8	1.8	1.8
Total	1000	1000	1000

R: control; UC: ulcerative colitis; RBE: ethanol extract of rice bran; ADE: ethanol extract of whole-grain adlay seeds.

**Table 2:** Scores of diarrhea, bloody stool, disease activity index, and colon length.

Group	Diarrhea	Bloody Stool	DAI	Colon Length (cm)
R	0 (0, 0) ^b^	0 (0, 0) ^b^	0 (0, 0) ^b^	6.93 ± 0.26
UC	2 (0, 2) ^a^	0.5 (0, 2) ^a^	1 (0.33, 1) ^a^	6.90 ± 0.38
RBE	0 (0, 2) ^ab^	0 (0, 1) ^ab^	0.67 (0.33, 1.33) ^a^	6.53 ± 0.16
ADE	0 (0, 0) ^b^	0 (0, 2) ^ab^	0 (0, 1.33) ^ab^	6.44 ± 0.19

Values are median (interquartile range, IQR) or mean ± SEM, n = 10−11 per group. DAI, disease activity index. Different superscript letters represent significant differences (Kruskal–Wallis test and Mann–Whitney U test, *p* < 0.05).

**Table 3:** Plasma levels of cytokines and chemokine MCP-1.

Group	TNF-α	IL-6	IFN-γ	IL-10	IL-12p70	MCP-1
	(pg/mL)					
R	38.4 ± 0.9 ^a^	53.4 ± 0.5 ^b^	12.00 ± 0.27 ^a^	409 ± 5 ^a^	106.8 ± 3.3 ^a^	10.05 ± 0.13 ^a^
UC	37.7 ± 3.2 ^a^	67.1 ± 8.8 ^a^	11.38 ± 0.29 ^a^	393 ± 4 ^a^	97.0 ± 3.9 ^a^	10.81 ± 1.29 ^a^
RBE	29.2 ± 1.3 ^b^	53.3 ± 2.5 ^b^	9.79 ± 0.17 ^b^	364 ± 5 ^b^	71.8 ± 4.2 ^b^	8.30 ± 0.21 ^b^
ADE	30.3 ± 1.8 ^b^	50.2 ± 0.9 ^b^	9.82 ± 0.45 ^b^	364 ± 4 ^b^	81.6 ± 4.7 ^b^	8.36 ± 0.32 ^b^

Values (pg/mL) are mean ± SEM, n = 10−11 per group. TNF, tumor necrosis factor; IL, interleukin; IFN, interferon; MCP, monocyte chemoattractant protein. Different superscript letters represent significant differences (one-way ANOVA and Duncan’s multiple range test, *p* < 0.05).

**Table 4:** Microscopic injury scores of the colon.

Group	Area of Ulceration	Edema	Immune-Cell Infiltration	Goblet-Cell Loss	Inflammation	Total Injury Score
R	0 (0, 0)	0 (0, 0) ^b^	0 (0, 0) ^b^	0 (0, 0) ^b^	0 (0, 0) ^b^	0 (0, 0) ^b^
UC	0 (0, 1)	1 (0, 1) ^a^	1 (0, 3) ^a^	0.5 (0, 1) ^a^	2 (0, 3) ^a^	4.5 (0, 10) ^a^
RBE	0 (0, 1)	1 (0, 1) ^a^	1 (0, 3) ^a^	1 (0, 1) ^a^	2 (0, 3) ^a^	6 (0, 9) ^a^
ADE	0 (0, 1)	1 (1, 1) ^a^	2 (1, 3) ^a^	1 (1, 2) ^a^	2 (1, 3) ^a^	8 (4, 11) ^a^

Values are median (IQR), n = 10−11 per group. Different superscript letters represent significant differences (Kruskal–Wallis test and Mann–Whitney U test, *p* < 0.05).

**Table 5:** Levels of reduced and oxidized glutathione and TBARS in the colon.

Group	GSH (μmol/g Colon)	GSSG (μmol/g Colon)	GSH/GSSG	TBARS (nmol/g Colon)
R	33.8 ± 2.0 ^b^	6.23 ± 0.44	5.60 ± 0.31 ^b^	132.5 ± 13.7
UC	43.9 ± 3.4 ^a^	5.49 ± 0.37	8.60 ± 0.50 ^a^	143.3 ± 29.6
RBE	30.0 ± 1.3 ^b^	5.60 ± 0.31	5.10 ± 0.39 ^b^	138.1 ± 22.6
ADE	35.3 ± 1.8 ^b^	6.63 ± 0.19	5.30 ± 0.30 ^b^	158.2 ± 25.0

Values are mean ± SEM, n = 10−11 per group. GSH, reduced glutathione; GSSG, oxidized glutathione, GSH/GSSG, ratio of the reduced to oxidized glutathione; TBARS, thiobarbituric acid reactive substance. Different superscript letters represent significant differences (one-way ANOVA and Duncan’s multiple range test, *p* < 0.05).

**Table 6:** Levels of cytokines and chemokine MCP-1 in the colon.

Group	TNF-α	IL-6	IFN-γ	IL-10	IL-12p70	MCP-1
	(pg/g Colon)					
R	31.2 ± 1.5 ^b^	8.92 ± 0.85 ^b^	2.08 ± 0.16 ^b^	3.08 ± 0.15 ^a^	56.8 ± 4.3 ^c^	0.25 ± 0.08 ^b^
UC	55.4 ± 4.1 ^a^	18.11 ± 2.84 ^a^	5.49 ± 0.37 ^a^	1.52 ± 0.14 ^b^	112.0 ± 8.9 ^a^	0.51 ± 0.10 ^a^
RBE	40.3 ± 3.3 ^b^	10.85 ± 2.57 ^b^	4.81 ± 0.28 ^a^	3.42 ± 0.20 ^a^	72.4 ± 9.2 ^b^	0.32 ± 0.08 ^b^
ADE	44.8 ± 2.6 ^b^	13.02 ± 1.99 ^ab^	4.92 ± 0.34 ^a^	2.15 ± 0.23 ^ab^	83.9 ± 6.7 ^b^	0.40 ± 0.17 ^ab^

Values are mean ± SEM, n = 10−11 per group. TNF, tumor necrosis factor; IL, interleukin; IFN, interferon; MCP, monocyte chemoattractant protein. Different superscript letters represent significant differences (one-way ANOVA and Duncan’s multiple range test, *p* < 0.05).
